# Acknowledgement to Reviewers of *Proteomes* in 2018

**DOI:** 10.3390/proteomes7010003

**Published:** 2019-01-08

**Authors:** Proteomes Editorial Office

**Affiliations:** MDPI, St. Alban-Anlage 66, 4052 Basel, Switzerland

Rigorous peer-review is the corner-stone of high-quality academic publishing. The editorial team greatly appreciates the reviewers who contributed their knowledge and expertise to the journal’s editorial process over the past 12 months. In 2018, a total of 50 papers were published in the journal, with a median time to first decision of 17 days and a median time to publication of 46 days. The editors would like to express their sincere gratitude to the following reviewers for their cooperation and dedication in 2018:

Achour, BrahimMartínez-Esteso, María JoséAl-Horani, RamiMartins, Ian JamesAryal, Uma KMatin, AbdulBalasubramanian, GaneshMeleady, PaulaBasavarajappa, Balapal S.Michiel, VermeulenBasit, AbdulMiki, YasuhiroBaucum, Anthony J.Mills, KevinBayés, ÀlexMilovanovic, DragomirBegun, JakobMiniaci, Maria ConcettaBiederer, ThomasMitulovic, GoranBocian, AleksandraModhiran, NaphakBothner, BrianMoro, LoredanaBurton, JeremyNagai, KouheiCampbell, CoreyNestler, UlfCanela, NúriaOkumura, NobuakiCarpentier, SebastienPadula, MatthewCash, PhillipPani, BibhusitaChen, Chieh-fuPasini, LuigiChen, Yu-JuPaulo, JoaoChen, ZhenPeppelenbosch, MaikelCheng, ZhePetyuk, VladislavChristians, UwePhilipp, StephanCiregia, FedericaRakus, DariuszCotella, DiegoRatajewski, MarcinCsősz, ÉvaReinders, JoergCuervo Escobar, PatriciaReisner, AndreasDehzangi, Abdollah (Iman)Richa, TambiDesselberger, UlrichRuss, BrendanDidonna, AlessandroSainlos, MatthieuDoblin, MonikaSancho, JaimeDodge-Kafka, KimberlySathe, Gajanan JalindarnathDoucette, Alan A.Sgarra, RiccardoDudley, EdShui, Hao-aiEhrnhoefer, DagmarShukla, HemFinnie, ChristineSiebert, StefanFischer, RomanSierecki, EmmaFricker, LloydSimonsen, Anja HviidGarg, PallaviSmalla, Karl-HeinzGastaldello, StefanoŠrámek, JanGrant, SethSrivastava, MugdhaGrider, ArthurStintzi, AlainGuo, MingquanStodghill, PaulHartman, Nathaniel W.Sudhir, Putty ReddyHong, QiutingTakáč, TomášHsuan, JustinTanca, AlessandroIqbal, Tariq H.Tao, WeiguoJagtap, PratikThenet, SophieJob, DominiqueTorregrossam, MaryJosic, DjuroTrim, CarolKalló, GergőUetz, PeterKanekiyo, TakahisaUhrig, R GlenKatselis, GeorgeVemula, HarikaKatsoris, PanagiotisWalsh, DavidKleiner, ManuelWang, XinwenKobeissy, Firas H.Wang, XueKrüger, MarcusWang, Yi-zhiLasonder, EdwinWilliams, Christopher C.Laura Capriotti, AnnaWoo, Sun HeeLepczynski, AdamXie, Zhong-RuLesner, AdamYang, Jae-WonLi, HuinanYu, XiaoboLuczak, MagdalenaZhang, YixiangLuthra, AmitZhang, ZhenbinMaász, GáborZhao, LiangMansuri, Shahid


